# Medicos, poultice wallahs and comrades in service: masculinity and military medicine in Britain during the First World War

**DOI:** 10.1080/23337486.2019.1677040

**Published:** 2019-10-14

**Authors:** Jessica Meyer

**Affiliations:** School of History, University of Leeds, Leeds, UK

**Keywords:** First World War, caregiving, masculinity, Royal Army Medical Corps (RAMC), medical officers, orderlies, stretcher bearers

## Abstract

The subject of British military medicine during the First World War has long been a fruitful one for historians of gender. From the bodily inspection of recruits and conscripts through the expanding roles of women as medical care providers to the physical and emotional aftermath of conflict experienced by men suffering from war-related wounds and illness, the medical history of the war has shed important light on how the war shaped British masculinities and femininities as cultural, subjective and embodied identities. Much of this literature has, however, focused on the gendered identities of female nurses and sick and wounded servicemen. Increasingly, however, more complex understandings of the ways in which medical caregiving in wartime shaped the gender identities of male caregivers are starting to emerge. This article explores some of these emerging understandings of the masculinity of male medical caregivers, and their relationship to the wider literature around the complex and sometimes contradictory relationship between warfare and medicine. It examines the ways in which the masculine identity of male medical caregivers from the ranks of the Royal Army Medical Corps, namely stretcher bearers and medical orderlies, was perceived and represented both by the men themselves and those they cared for. In doing so it argues that total war played a crucial role in shaping social and cultural perceptions of caregiving as a gendered practice. It also identifies particular tensions between continuity and change in social understandings of medical care as a gendered practice which would continue to shape twentieth-century British society in the war’s aftermath.

In 1907, the *British Journal of Nursing* published an article announcing the foundation of ‘a society to be known as “The Navy and Army Male Nursing Co-operation”, the object being to enable First Class Orderlies to find employment in civil nursing upon leaving the Service.’ (The Navy and Army Male Nursing Co-operation 1907). The purpose of this body was not simply to provide employment for a specific group of ex-servicemen, but also to fulfil a perceived need, ‘more generally acknowledged than supplied’, for ‘properly trained [men] who can be trusted in emergencies to lend assistance, or who can be given responsible charge of special cases’ in the provision of long-term nursing care to men.

The origins of the Co-operation appear to lie in the 1907 Haldane Reforms to the military, including the military medical services, which were, in turn, a response the perceived strategic and tactical disasters of the 2nd Anglo-Boer War (1899–1901) (Spiers 1980). The outbreak of the First World War seven years later reinvigorated the organization, when its mission was increasingly cast as one of gender sensitivity, based on a clear understanding of separate gender roles within medical caregiving. As Sir Dyce Duckworth, the Co-operation’s president, argued, ‘There would always be cases of disease and ailment for which men alone were appropriate [as nurses]. There were certain cases not fit to be nursed by women, and a great many men objected to being nursed by women’ (The Navy and Army Male Nursing Co-operation 1915). Lady Tree, a patron of the Co-operation, told one newspaper that ‘she had been privileged to visit lately many military hospitals, and she had always been struck by the extraordinary tenderness and gentleness of the Army male orderlies. It seemed to her that a man could go on tip toe far more softly than could any woman … and she believed that in nursing the touch of a man was much more gentle than was that of a woman.’ (The Navy and Army Male Nursing Co-operation 1914).

Despite the case made by the Co-operation into the early 1920s, that a specific organization for the employment of male nurses was needed because men who were ‘seriously disabled [in the war] … would prefer as nurses a male comrade who had served in the great war to a civilian who had been in England the whole of the past four years’ (The Navy and Army Male Nursing Co-operation 1920), this particular group appears to have become defunct by the middle of the decade. However, its existence throughout the years of the First World War points to an important facet of medical caregiving in wartime, the role of male caregivers in providing medical care to men injured and disabled in war. Much of the historical literature around British medical care in the war has focussed either on the technical and organizational developments in treatment and evacuation (Harrison 2010), on the evolving roles of female nurses as wartime caregivers (Hallett 2009; Watson 2002; Fell and Hallett 2013), or on the impact of war injury and illness on the identities of soldier patients (Carden-Coyne 2009; Gagen 2007). All these analyses show how the war shaped caregiving as a gendered practice for both providers and recipients. Yet a great deal of caring work was undertaken by men in wartime, both the medically trained officers and the non-commissioned servicemen of the Royal Army Medical Corps (RAMC), including the stretcher bearers, ambulance drivers and medical orderlies who the Navy and Army Male Nursing Co-operation sought to find employment for as trained caregivers in the war’s aftermath. As the comments of Sir Dyce Duckworth and Lady Tree indicate, the work that these men undertook in their war service was gendered in distinctive ways.

In this article, I will argue that the complexities and contradictions inherent in the provision of military medical care in the context of total war were as significant in shaping the gendered identities of male caregivers as they were for female nurse and wounded patients. Using a range of forms of historic cultural production, including newspaper articles, hospital journals and published memoirs, I will look at representations of different groups of men who engaged with military medical care specifically as providers to show how the role of medical caregiver shaped a unique masculine identity in wartime, one that was defined by issues of class, autonomy, professionalism and bodily integrity. In particular, I will examine the men of the RAMC, both trained medical officers and the Other Ranks of the Army Medical Services (AMS), the stretcher bearers and medical orderlies who staffed the line of evacuation from fighting line to home front, to show how these men’s wartime masculinity was shaped by the work they undertook and, in turn, how their work shaped cultural perceptions of military medical care as gendered practice.

To do this, I will first examine the existing literature on British wartime medical caregiving to demonstrate how this historiography has developed useful interdisciplinary methodological approaches at the intersection of cultural, social and medical history. In particular, the practice of close reading verbal and visual sources alongside the analysis of political and professional literature will be shown to underpin scholarship in the field. However, while this methodology has been utilized to develop nuanced understandings of the work of particular categories of caregiver, including nurses and doctors, as well as the experience of patients, the roles and status of non-professional male caregivers in wartime have yet to be fully explored in this way. The article will therefore turn to a more in-depth analysis of these men, examining how the work of stretcher bearers and nursing orderlies can be compared to that of military medical officers. By exploring a range of contemporaneous cultural representations of these men across rank and role, I will demonstrate how the better understanding of their place in a society mobilized for total war enhances our knowledge of medical care as gendered practice in the first half of the twentieth century.

## War, medicine and gender

Both histories of gender and histories of medicine during the First World War have used the physical and psychological depredations wrought by the conflict on the body as a lens through which to explore the impact of war on both social and subjective experience. Whether examining official records of military medical practice, cultural representations of caregiving, or the personal records of carers and patients, such studies have pointed to the ways in which total war used and abused gender and medical care provision to exert control over individuals caught up in its totalizing logic. For example, Joanne Bourke’s study of medical inspections of the male bodies of volunteers and disabled ex-servicemen demonstrates how the male-gendered body was assessed and categorized with the aim of rationalization and efficiency (Bourke 1996). Similar motivations underpinned the development of triage practices as discussed by Mark Harrison (Harrison 2010). Elaine Showalter, meanwhile, in her pivotal classification of psychological wounding during the war as a form of ‘male hysteria’, argues that the location of gendered definitions of ‘shell shock’ within a wider context of medical care as an abusive practice developed against women in peacetime was a method by which the military hierarchy exerted control over the mass of the lower ranks of service personnel. (Showalter 1987). Other works in this field have shown how the physical wounds of war and their treatment influenced constructions of gender and gender difference throughout British society (Carden-Coyne 2009; Gagen 2007).

Such studies tend to present the relationship of the wounded and disabled men, the ‘poor brave souls’ of charitable campaigns (Cohen 2001, 101–148), with wartime medicine as either an effeminizing or infantilising one (Jones 2014, 417). The shell shock sufferers of Showalter’s analysis ‘were silenced and immobilized [by war] and forced, like women, to express their conflicts through the body.’ (Showalter 1987, 171). By comparison, the psychologically traumatized men treated by Dr William McDougall were viewed as suffering from ‘regression’, ‘an actual re-animation of the dispositions [of infantile modes of behaviour] that had been latent or in suspended animation’ (McDougall 1920, 137). In cases of physical war wounds, Seth Koven has shown how amputees were compared to disabled children in the publicity literature of both the government and charities, an association which benefitted children but had more ambiguous outcomes for the war-disabled man whose claims to mature masculinity were cast into doubt (Koven 1994).

More recent analyses have, however, complicated these readings. Even some texts that hinge on the idea that the injured male body was constructed as problematically gendered by both society and individuals point to ways in which war wounds could be interpreted as appropriately masculine. Thus Ana Carden-Coyne, in *The Politics of Wounds*, argues that the donning of hospital ‘blues’ both ‘suggested that underneath the clothes was dysfunctional, emasculated flesh’ and ‘signalled the heroism of wounded men, aiding their capacity to strike up romances with local women’ (Carden-Coyne 2014, 215, 217). In this she builds on Jeffrey Reznick’s argument that hospital ‘blues’ emasculated military patients by not including pockets even as they ‘served an important propagandistic function during the war, helping to put the wounded Tommy on public display and facilitate public appreciation of his service to King and Country.’ (Reznick 2004, 103–105). Wendy Gagen, meanwhile, has demonstrated how, in the case of J. B. Middlebrook, individual experiences of wounding and disablement could be negotiated in ways which allowed subjective understandings of the self as appropriately masculine to be maintained. The subjective struggles that Middlebrook faced in maintaining this self-perception, however, highlight the important interface between bodily and psychic trauma which was central to the gendering of the war wounded (Gagen 2007). The literature relating to the masculinity of the war wounded and disabled has thus developed considerable nuance over time. The embodiment of gender identity remains, however, the central tenet of this field. As Gagen concludes, ‘For disabled men, a masculine identity was necessarily rooted in corporeality, even if it aimed to transcend or even control it.’ (537). Yet in centring on the body, these analyses reinforce the idea that the contestation of masculinity within the context of medical caregiving relates to men as objects of care rather than contributors to practice.

By comparison, it has been the female nurse who has tended be the focus of studies of gender in relation to the role of caregiver in this period. This in part reflects the dramatic changes in interactions between women and military institutions in Western Europe and America in the second half of the nineteenth century, identified by Barton C. Hacker (Hacker 2012). In Britain, Hacker traces these changes to the Crimean War, the conflict that Holly Furneaux also identifies as the point when ‘The national shame produced by the insufficiencies of army medicine combined with gendered and classed ideals of women as curative, ministering angels to invalidate soldiers’ work in military hospitals.’ (Furneaux 2016, 216). As a result of cultural representations of this understanding, the British military nurse became, in Anne Summers’s words, ‘both a real and imagined woman’ in the era preceding the First World War (Summers 1988, 1). At once a community of trained women seeking to establish their identities within social and military hierarchies, and a cultural ideal used for propagandistic purposes to exert gendered hegemonies over men and women alike, British military nurses in the second half of the nineteenth century and the early decades of the twentieth have proved a rich source for understanding the impact of war, which Joan W. Scott terms ‘the ultimate disorder, the disruption of all previously established relationships’ (Scott 1987, 27), on gender relationships in particular.

Much careful scholarship has helped unpick this complex and conflicting image of war nurses in recent years, demonstrating how the practice of medical caregiving was an important area of gender definition in this period. Christine Hallett and Janet Watson, for instance, have both shown how wartime experiences shaped the identities of trained nurses as professional women, in comparison with VAD constructions of a voluntarist wartime identity morally equivalent to the male combatant volunteer (Hallett 2009; Watson 2002). Alison Fell, meanwhile, has shown how nurses mobilized gendered narratives to construct heroic caregiver identities in both wartime and its aftermath (Fell 2018). Beyond these discussions of how a masculinized wartime culture shaped the identities of female nurses as a class, Santanu Das has explored how nursing roles could conflict with social norms of gendered caring in ways which expose an ‘alternative history of war trauma’, one which is explicitly gendered as female (Das 2005, 194). Margaret Higonnet similarly argues that nurses’ narratives ‘explore the dilemmas facing frontline nursing, where self-control and technical efficiency conflict with emotional involvement and the threat of hysteria.’ They enable ‘the reader to weigh the competing values engaged in the practice of military medicine’ (Higonnet 2001, xix), even as they identify those values as particular to women and distinct from the rival imperatives which so immobilized the traumatized men of Showalter’s analysis.

More recently Carol Acton and Jane Potter have sought to expand and complicate this reading of war trauma by incorporating the narratives of other wartime medical caregivers, principally doctors, across twentieth-century conflicts (Acton and Potter 2015). While this approach nuances readings of medical subjectivities in wartime, the foregrounding of trauma in all these analyses ensures that the gendering of care provision maps on to the gendering of sick and wounded, creating potentially over-neat dichotomies of masculinity and femininity as they relate to the power structures of care provision. Carden-Coyne, meanwhile, has presented a sophisticated analysis of the complex relationships between gender, care provision and power in hospital cultures by exploring the multifarious social and cultural interactions between the gendered patient and gendered carer. Yet even here the gendering of the doctors and orderlies who formed the male staff of such institutions receives comparatively little attention, particularly in terms of their relationships with patients as gendered subjects. Rather, the analysis of relationships between these men that Carden-Coyne draws out relates to issues of race, nationality and class (Carden-Coyne 2014). Given John Tosh’s contention that masculinities in Britain in the late nineteenth and early twentieth centuries can only be understood if examined in the round, that is, in the context of work and homosocial spaces as well as the domestic (Tosh 1994), a significant area of analysis in relation to male caregivers during the war remains to be fully explored.

## Medicos

One of the risks of using female nurses as the primary route into exploring caregiving as a gendered practise in wartime is the potential for such analyses to implicitly reinforce nineteenth century beliefs that women have a privileged perspective on care provision *because* they are women. Yet the dilemmas identified by Higonnet in the writings of nurses such as Mary Borden and Ellen La Motte are equally present in the writings of male doctors serving in the military medical services. Similar personal writings by men have been the subject of scholarly enquiry by historians of medicine, although not necessarily using the lens of gender, as has almost always been the case with nurses’ narratives. The medical and military imperatives placed on medical officers to heal wounded men professionally and efficiently, with the primary aim of returning them to the battlefield to both inflict harm on the enemy and face further harm themselves, often came in to conflict. Men such as W. H. R. Rivers found themselves deeply troubled by these contradictions (Slobodin 1978). While not all medical officers approached their work with Rivers’s sensitivity to its potential paradoxes, wartime recruitment, as Ian Whitehead has shown, posed direct challenges to the often precarious professional integrity of British doctors: ‘Early in the War the influence of Sir Alfred Keogh and the links between the civil and military branches of the profession helped to ensure that there was an awareness of the need for civil-military co-operation. However, as the drain on medical manpower became more acute, goodwill alone could not prevent growing suspicion that the Army’s employment of doctors was extravagant.’ (Whitehead 1999, 85). Bourke, Carden-Coyne and Leo van Bergen, meanwhile, have all demonstrated how the identity of doctors as providers of care was threatened by soldiers’ perceptions and representations of their brutality, in part a reaction to their role in policing behaviour and enforcing military discipline (Bourke 1996; Carden-Coyne 2014; van Bergen 2009).

All these challenges can be seen to have implications for understandings of the specifically masculine identity of doctors in wartime. The ambivalence of men like Rivers to the war had the potential to throw into question their sense of both patriotic and professional duty, ideas which underpinned concepts of male citizenship in peace and war. (Gullace 2002). The struggles between military and medical authorities over the recruitment, training and roles of male doctors in wartime explored by Whitehead threw open to question these men’s professional identities even more directly. Were they soldiers or civilians? Was their first duty to the military hierarchy, in which they served as officers, or their patients as care providers, even if often authoritarian ones? How much autonomy could they lay claim to in prescribing treatments and providing diagnosis? As both doctors and servicemen, these men held positions which gave them access to important forms of masculine authority, particularly over young working-class rankers, through officer status and access to professional expertise (Meyer 2009b). At the same time, they occupied wartime roles as non-combatants; the RAMC was a non-combatant unit under the terms of the 1864 and 1906 Geneva Conventions and, as such, its members, including its officers, were not allowed to carry arms except in their own defence and that of their patients. In a society engaged in total war, where combatant status was celebrated as the epitome of masculine expression (Ugolini 2013, 7), this status, especially when allied with war work increasingly associated with women (Hacker 2012), cast the wartime masculinity of doctors into doubt. The extent to which these men were viewed as suspect in the eyes of the military authorities can be seen in General Garnett Wolseley’s order at the Royal Review in Phoenix Park, Dublin in 1900, that ‘those medical people … return their swords. Inform them that they are only civilian attendants upon sick soldiers’ (Blair 1998, 42). As Whitehead has demonstrated, such attitudes persisted, not only in the years before 1914 but also throughout much of the war. (Whitehead 1999, 15–16). As a result, the reputation of medical officers within military ranks, was, like that of military chaplains (Madigan 2011) not high.

In addition to the perception that their role was unsoldierly, the status of medical officers was further undermined because the ordinary soldier’s primary contact with these men was in the role of military detective, always on the lookout for malingerers, and disciplinarians, doling out medicine and duty rather than care and sympathy (Bourke 1996, 89–94). This provided scope for resentment and mockery which served to undermine medical officers’ authority, not only with regards to Regimental Medical Officers conducting daily medical parades, but also those providing care in hospitals further down the line (Carden-Coyne 2014, 309). Indeed, in van Bergen’s view, medical caregivers of the First World War never fully recovered either subjectively or reputationally from the brutality that war inflected on them and, in turn, caused them to inflict on others (van Bergen 2009, 401–405). Certainly, in canonical literature published after the war, medical officers were often portrayed as brutal, violent, uncaring and frequently drunk. Wilfred Owen’s poem ‘The Dead Beat’, for example, closes with the image of ‘the Doc’s well-whiskied laugh:/“That scum you sent last night soon died. Hooray!”’ (Owen 1963, 72). Max Ploughman’s encounter with another medical officer is described in equally damning terms:

I find [Brown] lying on the ground, breathing heavily and apparently unconscious. … This is a case for the doctor. After searching for some time, I find him at mess in the Headquarters dug-out; so I send down a message. He comes up, evidently annoyed at being disturbed, so I apologise as we go to the boy together. The doctor bends over him a moment, and then, rising, shouts with astonishing fury: ‘You damned young scrimshanker, get up! What the devil do you fancy you’re playing at? Think you can swing the lead on me? Get up, or I’ll have you in the guard room.’ He pushes the boy with his foot, but the lad does not stir. ‘Don’t you think he is ill?’ ‘Ill? There is nothing the matter with him at all. Just “wind up”, the bloody young coward. Leave him there if he doesn’t get up, and don’t call me again. I don’t waste my time over these damned scrimshankers.’ He turns and goes back to the dug-out. This strikes me as callous brutality, and for a moment I am at a loss to know what to do. The men around come to the rescue. They pick the boy up, assuring me they will look after him. As they carry him off, I hear them murmuring, ‘Brute.’ ‘Swine.’ (Plowman 1927, 190).

Van Bergen’s judgement, which covers medical professionals of all ranks and on all sides of the conflict, is sweeping and may have more relevance in the German than in the British context (Perry 2014, 6–7). However, doctors sitting on the medical boards which assessed disabled ex-servicemen for war disability pensions in Britain from 1917 continued to earn a reputation for judgemental unfairness in their treatment of disabled ex-servicemen, reflecting the extension of their wartime role as medical policemen, a status that saw them caught between professional imperatives to care and political insistence on financial prudence. During the conflict, however, many regimental medical officers, and even surgeons based at Casualty Clearing Stations and hospitals along the line of evacuation were able to salvage some of their reputation and retain their authority when they served under fire, just as many chaplains were able to do (Madigan 2011). Medical officers such as Noel Chavasse, who earned one of two double VCs awarded to RAMC officers during the war, were viewed by combatants as particularly heroic, not only because they were sacrificing themselves in the cause of caring for others, a narrative which fit particularly neatly with prewar constructions of self-sacrificial Christian manliness (Meyer 2009a, 80), but also because they faced danger and death without the possibility of fighting back. The psychological disadvantage that this gave the men who served in the unit in battle was widely acknowledged, and generally deemed a signifier of their particular and unique courage. As A. E. Francis commented in his history of the 2nd/3rd East Lancashire Field Ambulance, it was one thing ‘to do gallant deeds with arms in hands and when the blood is up but the courage demanded to walk quietly into a hail of lead to bandage and carry away a wounded man, that is worth talking about.’ (Francis 1930, 87).

## Poultice wallahs

As Francis’s point about carrying away wounded men under fire indicates, it was not only the officers of the RAMC who faced the enemy under fire without arms. The men who served under them, as stretcher bearers and nursing orderlies, did also. Like military doctors, they produced a range of personal narratives in the form of letters, memoirs and cultural artefacts such as hospital journals. This section and the next examine this cultural material to explore its representation of these men’s subjective understanding of their roles and status as male caregivers in wartime.

Like doctors, who formed the officer corps of the RAMC, the stretchers bearers and orderlies who made up the ranks of the Corps found that their masculine identity was challenged by their wartime service. Like medical officers, they were faced with competing imperatives. Although they served in uniform, and under military discipline which curtailed their autonomy, they were, as has been noted, non-combatants. In the popular press, they were often accused of being ‘slackers in khaki’, men who avoided the dangers of battle while claiming the martial status with which a uniform endowed men in wartime (Muir 1917, 147–158). Within the military itself, many like George Swindell found themselves labelled with derogatory epithets such as ‘Rob All My Comrades’ and ‘Run Away, Matron’s Coming’ (Swindell n.d). The first of these epithets reflects the practical fact that an RAMC stretcher bearer was often the last man seen handling a wounded man’s personal effects, which were then lost in the evacuation process. The second, however, points to a gendered reading of the orderly’s roles as cowardly and emasculated due to their subservience to female nursing staff (Meyer 2019, 47–48, 161–162). This was made explicit in the pages of the *Gazette of the 3rd London General Hospital* (hereafter the *Gazette*), in which the hospital orderly was depicted as ‘a gentle hen-pecked spouse’ and that most Victorian of female figures, ‘an Angel in the House’ by one satirical poet. (Balladmonger 1918).

**Figure 1.**
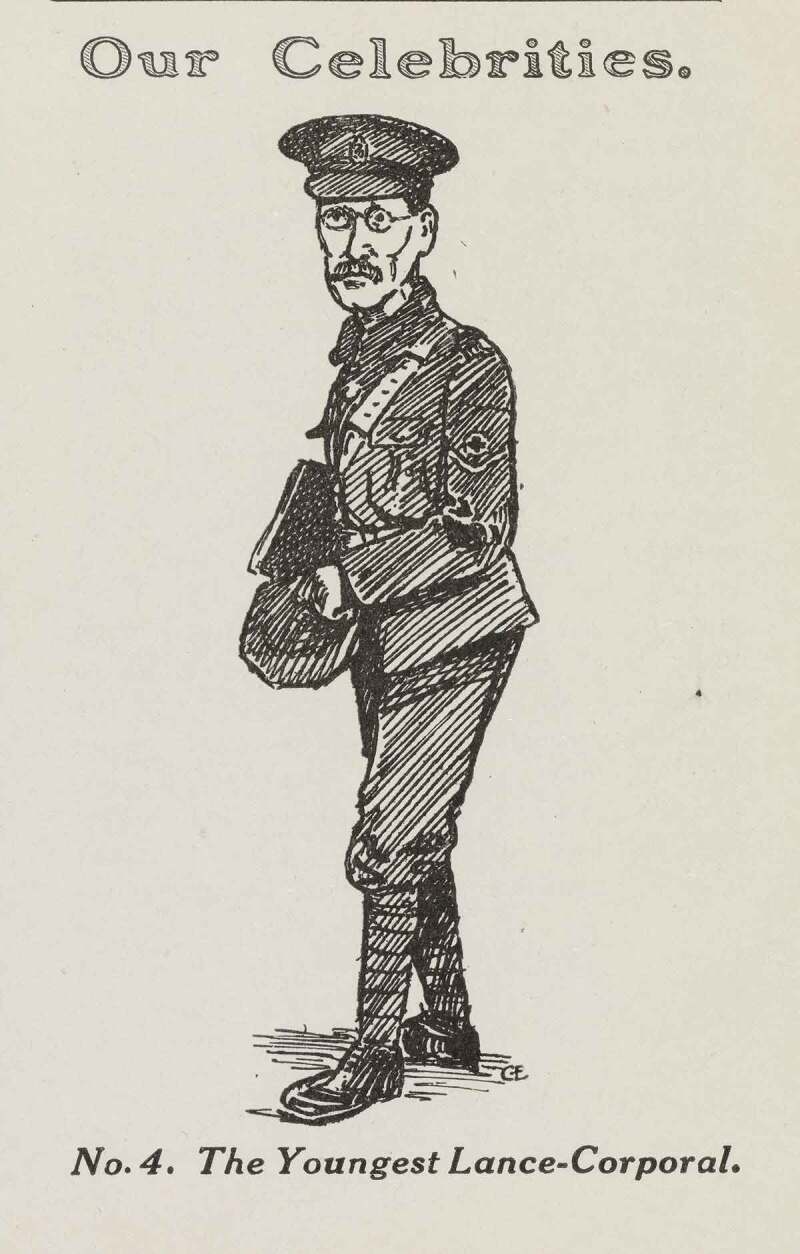
'Our Celebrities, No. 4: The Youngest Lance-Corporal', *Gazette of the 3rd London General Hospital* (December 1915)

These representations of RAMC rankers’ cowardice and disloyalty, both in military and gender terms, were not aided by the fact that military recruitment policy towards the medical services meant that those who served in the RAMC were often physically frail, overaged, or otherwise ‘unsoldierly’ in body. A satirical article in the *Gazette* described the stereotypical male orderly at the hospital as ‘a little, insignificant looking man, with sparse hair and a heart that insurance companies wouldn’t bet on. He also suffered from asthma and a dictatorial wife.’ (Doré 1918). A cartoon in the same journal depicted him in a similar manner (Figure 1).

As the war went on, rather than drawing on recruits and conscripts with physical classifications of A1, who were instead channelled into combatant units, the ranks of the RAMC were supplemented by those of B1 class or below (C1 for those on home service), both new recruits and those left with the long-term effects of previous wounds or illness (Macpherson 1921, 138–140). The ranks of the unit were also, although to a lesser extent, supplemented by conscientious objectors, men who were willing to serve their country in a capacity that did not involve the taking up of arms or violence towards the enemy. This last group of men, as Lois Bibbings has shown, were commonly portrayed as unmanly, morally as well as physically (Bibbings 2009). Newspaper stories focussing on the military tribunals which ruled on appeals against conscription regularly reported on those willing to serve with the RAMC rather than in roles involving the bearing of arms or taking of life (McDermott 2011, 36). They also commented on those assigned to the Friends Ambulance Unit, a voluntary unit which came to an agreement with the War Office ‘to collaborate in arranging work of “national importance” for members of the Society coming under the Act; so that all Quakers coming before the tribunals to be set up for conscientious objectors could be referred to the Friends Ambulance Unit.’ (Ormerod Greenwood 1975, 181). The association of these men with the medical services in the public mind had a potentially damaging effect on the overall image of RAMC servicemen as appropriately masculine.

Yet many RAMC rankers managed, over the course of their war service, to negotiate the seeming contradictions of their service, constructing subjective masculine identities that accommodated both negative connotations and more positive roles. The *Gazette* was edited and, in many instances, written by the RAMC orderlies of the hospital, many of whom were members of the Chelsea Arts Club (Muir 1918). The negative images of RAMC ranker masculinity it contains can, therefore, be read as ironic appropriations as much as a pointed critiques of insufficient manliness. More positively, men serving overseas, as with regimental medical officers, were able to demonstrate their courage by going unarmed into battle, placing their bodies at risk in order to clear and treat the wounded of both sides. On 27 November 1914, David Randle McMaster, a nursing orderly with the 24th Wessex Field Ambulance, wrote a detailed description of his first experience of stretcher bearing in the front line during which he came under fire, concluding ‘Thus ended my first experience of rifle fire, but I don’t expect it will be the last by a long way.’ (McMaster 1914). All along the chain of evacuation stretcher bearers and nursing orderlies attempted to counter images of physical frailty with equally potent ones of strength and endurance, whether through long carries in relays over periods of up to 24 h with little food and water (Chase 1924; Midwinter 1933), or the physical and emotional labour which ensured the efficient running of aid posts and hospitals. As one poem in *The ‘Southern’ Cross*, journal of the 1st Southern General Hospital, Birmingham, put it:

Oh! it’s weary work in the white-washed ward,
Or the blood-stained Hospital base,
To number the kit of the man who was hit
And cover the pale, cold face,
Or hand out fags to the brave boys in rags,
Who’ll stick it and cheerfully grin,
As the deftly used knife cheats grim death of a life
While the grey of the dawn creeps in.
To hold the hot hand of the man who talks wild
And blabs of his wife or his kids,
Who dreams he is back in the old home again,
Till the morphia bites, and he loses his pain
As sleep settles down on his lids.
The ‘Hospital Orderly’ doing his bit,
Of V.C.s not many they score,
Yet are earned every day in a quiet sort of way
By the ‘Royal Army Medical Corps.’ (Atkins 1917)

In the process of providing care, many of these initially untrained men serving overseas developed levels of knowledge first aid and medicine which allowed them to lay claim to their own sense of semi-professional wartime identity. George Swindell recalled ‘studying in our spare time, the R.A.M.C. manuals, to teach, and refresh our memories, how to carry and bandage wounded’ (Swindell n.d., 77), while J. B. Bennett, an office clerk who enlisted in 1914, noted in his memoirs that ‘It was routine to record on a clinical chart for each case, temperature, pulse, respiration, and bowels before [the patient was] seen by the Medical Officer, and when pressure was on most orderlies were competent in recording the first three items simultaneously and within two minutes.’ (Bennett n.d., 34). Both Swindell and Bennett were proud of the medical skills they had developed in their roles as RAMC rankers. In Swindell’s case, he used this new quasi-medical identity to directly challenge the classification of himself and his comrades by combatants as non-combatant ‘base wallahs’ (Swindell n.d., 90).

## Comrades in service

Even for men whose medical service placed them firmly out of harms way, there were opportunities to construct a sense of appropriate masculine identity, even as non-combatants in wartime. This identity was located firmly in their status as service personnel serving with a military unit, a role which allowed them to positon themselves as comrades to the combatants they cared for. Ward Muir, who served throughout the war as an orderly at the 3rd London General Hospital in Wandsworth, wrote of the injustice of the public spurning the ‘Bluebottles’, civilian ‘amateur’ members of the London Ambulance Corps, who wore distinctive blue, non-military uniforms, and whose selflessness in volunteering he praised. Yet he was quick to point out the importance of a distinctly service identity to RAMC servicemen. ‘However newly enlisted he is, the C3 youth who wears the Red Cross is a professional, and, consciously or otherwise, comports himself as such.’(Muir 1918, 102–103). However limited, repetitive and domestic his work might be, the home hospital orderly emerges from the pages of the *Gazette* and the two collections of essays which Muir published over the course of the war as a *uniformed* serviceman devoted to his duty. As Muir noted of the way enlistment shaped the home hospital orderly’s sense of self:

All he knew was that, by the quite simple process of putting on a khaki suit, he suddenly found a calm which he had not experienced for one minute since the war’s outbreak. The deed was done. He had enlisted. Scarcely in the way he had meant; but still, he had enlisted: he was genuinely in the army: not as the heroic Tommy Atkins of the battlefield, but as an unmistakable Tommy Atkins all the same, with a number, and a separation allowance, and a ‘Religion’ (Muir 1918, 17–18).

Despite his age and health which, as we have seen, cast doubt on his claims to wartime masculinity, the orderly needed physical strength and stamina to carry stretchers, bedding, food, water, wood and anything else loaded on him. Muir pointed out that ‘Without presuming to compare either the importance or the onerousness of the hospital orderly’s work with that of the soldier capable of going to the front to fight, I would here add that the critic who watches the stretcher-carrying party [outside a home hospital] and thinks it a pity that able-bodied males should be wasted on it, is doing the system (not to mention the men themselves) an injustice. For the men whom he sees are not, as a matter of fact, able-bodied, even though muscular enough to stand this short physical effort’ (Muir 1917, 102–103). He went on to note, however, that due to the labour of these same stretcher bearers, ‘I have known six hundred patients enter the hospital in forty-eight hours.’(148).

The home hospital orderly could even lay claim to what Plowman identified as the greatest quality of courage in men in the field, that of ‘caring for your pals more than yourself’. ‘Being there’, he declared was ‘the very basic test of manhood.’ (Plowman 1927, 100–101). While orderlies were not there in moments of danger and wounding as bearers could be, they could ‘be there’ for fellow servicemen in moments of pain and fear, from the dressing station to the operating theatre and even into the home, a space made strange and potentially fearful by permanent injury. One of Muir’s most moving columns tells the story of how he accompanied a blind man home by train upon his hospital discharge, a journey where Muir’s eyesight was a necessary adjunct to his charge’s sense of familiarity and homecoming (Muir 1917). The fact that orderlies were there was much appreciated by the men they cared for. As one patient at the 3rd London General wrote in the *Gazette*, the hospital ‘is kept by patriots who, through some cause or other, are unable to fight but do their bit by looking after those who have fought, and they do it well. Nothing is too big for them to attempt, and no detail is too small to receive attention. They do everything in their power to ease the sufferings of their brothers who have given their best for their country.’ (Kelk 1917). The writer was happy to acknowledge hospital orderlies’ claims to a shared sense of service and duty and, therefore, a patriotic masculine identity.

## Conclusion

In one of the essays in his 1918 collection *The Happy Hospital*, Muir offered ‘ironical thanks’ to the Kaiser on behalf of ‘some of us, weaker brethren … for having taught us that we are stronger than we supposed.’ (Muir 1918, 120). As this comment indicates, over the course of the war, male medical caregivers from the ranks of the RAMC laid claim to a sense of masculine identity that could be viewed as appropriate in the context of a society engaged in total war. Unlike the medical officers they served under, who could lay claim to a professional identity as trained doctors, a requirement of their rank, the stretcher bearers and orderlies of the RAMC were distinguished more by their physical insufficiency as military men than by any claims to expertise. Yet, like doctors, they sought to challenge cultural assumptions about the natural dominance of women as providers of care which reinforced negative social constructions of the male caregiver in wartime. Drawing on ideals of duty and comradeship, particularly as demonstrated by a willingness to serve under fire and the symbolism of shared military uniform, both officers and rankers of the corps strove to identify themselves as acceptable comrades in service rather than brutal bullies or cowardly ‘bandage wallahs’.

Not all such constructions were successful. While men like George Swindell and J. B. Bennett could record with their wartime service with pride in (unpublished) postwar memoirs, the reputation of the medical officer suffered in representations in published memoirs of men like Max Plowman. The dominance of the female nurse as an appropriate caregiver for wounded men was also enhanced by their wartime work, reflected in the Male Nursing Co-operation’s need to articulate the claims for male nurses to this role and its demise as an institution soon after the end of the war. Yet the fact of the Co-operation’s existence at all points to the multiple understandings of the role and status of male medical care providers in the era of the First World War, understandings which would continue to complicate social and cultural perceptions of caregiving as a gendered practise throughout the interwar period.

Looking at men associated with medical care as providers, therefore, exposes a range masculine identities far more diverse than the insufficient masculinities of men whose bodily integrity was threatened by wounds and disabilities, resulting in infantalization and effeminization. The masculinities of both medical officers and rankers during the war were inflected by ideas of military courage and medical professionalism, as well as being critiqued by anxieties over physical and moral frailty. Exposing the nuances of these men’s identities enables us to more fully read wartime caring as a gendered practice in this period. Viewed alongside nurses’ constructions of wartime identities, the work of male medical caregivers highlights both the range of labours involved in caregiving and the multiplicity of competing imperatives faced by all caregivers, and the ways in which these imperatives were often gendered in wartime culture and society. In particular, the physical labour undertaken by RAMC rankers as a part of their caring roles demonstrates the limits of women’s penetration of the conflict zones during the war. At the same time, the emotional labour orderlies undertook in hospitals more fully demonstrates the extent to which women’s expanding roles shaped contemporary interpretations of caregiving in terms of gender hegemonies. Orderlies’ ability to construct their work as part of a military masculine identity challenged cultural constructions of care as women’s work, even as it presented an alternative reading of appropriate military masculinity in a society engaged in total war. Undertaking emotional labour under the authority of trained women could be not merely acceptable for such men, but used by them to construct a distinctive quasi-professional identity of their own as both carers and wearers of military uniform. That men of the ranks of the RAMC actively sought to lay claim to such an identity and defend it through humour and in memoir points to the power of gendered hegemonies of caregiving and wartime service. These hegemonies were equally important in shaping the experiences of other categories of caregiver, but they emerge most clearly through comparisons across social and cultural categories.

Exploring the wartime identities of male care providers therefore enables us to more fully understand the impact of total war on medicine as both provision and practice. In particular, it points to the continuations in social and cultural understandings of medical care which underpinned many of the progressive and modernizing developments identified by historians of both medicine and gender. This is not to argue that such continuities contradict the progress made in women’s access to medicine as a professional role or the moves towards increased provision and rationalization of medical care by the State. Rather, identifying the tension between continuity and change in the social understanding of medical care as gendered labour during the war enables a fuller and more nuanced understanding of how care provision developed in the years after the war, laying the groundwork for the eventual creation of the welfare state. Understanding the roles of the doctors and men of the Royal Army Medical Corps as men providing care is thus a necessary part of the history of both the First World War and its legacies for British society.
